# Placental Adaptations Under Severe Anemia: Neonatal Outcomes at the Crossroads

**DOI:** 10.7759/cureus.110450

**Published:** 2026-06-08

**Authors:** Monika Gautam, Sushila Chaudhary, Sumiti Gupta

**Affiliations:** 1 Obstetrics and Gynecology, Pandit Bhagwat Dayal Sharma Post Graduate Institute of Medical Sciences, Rohtak, IND; 2 Pathology, Pandit Bhagwat Dayal Sharma Post Graduate Institute of Medical Sciences, Rohtak, IND

**Keywords:** low birth weight, placental examination, placental fibrosis, placental histopathology, preterm, severe anemia, syncytial knots

## Abstract

Anemia is a major global health problem, particularly in developing countries such as India, due to the greater prevalence of dietary iron deficiency, hemoglobinopathies, macronutrient deficiencies, and infections such as malaria, human immunodeficiency virus (HIV), and hookworm infestation. Anemia-induced hypoxia triggers a range of vascular and structural changes in the placenta, potentially leading to complications for both the mother and the fetus. Studies have shown that placentae from anemic mothers often exhibit hypertrophy, characterized by increased placental weight relative to fetal weight. Placental infarcts and calcifications occur due to inadequate blood flow, leading to areas of necrosis within the placenta.

To study these placental alterations and their corresponding correlation with maternal and fetal health, an observational prospective study was conducted at Pandit Bhagwat Dayal Sharma Post Graduate Institute of Medical Sciences, Rohtak, comprising two groups: Group A - severely anemic pregnant females and Group B - nonanemic pregnant females, each consisting of 40 individuals. The placentae from the two groups were first grossly examined and then sent for histopathological examination, and the findings were compared between the two groups. Neonatal outcomes were also correlated with these findings.

Our study found significant changes in the placentae of the severely anemic group in terms of an increased incidence of syncytial knots, placental fibrosis, placental calcifications, and villitis, as well as a reduction in placental weight and cord length. Placental fibrosis, calcification, and villitis were associated with adverse fetal outcomes such as preterm delivery and low birth weight.

The study concluded that microstructural changes in the placenta due to anemia are a significant contributor to premature and low-birth-weight infants.

## Introduction

The placenta is a vital organ of fetal life that is easily accessible and can be examined without any discomfort to the mother or the neonate. The placenta, being in close association with the developing fetus throughout gestation and exposed to conditions similar to those of the fetus, holds an estimated record of an infant’s prenatal timeline.

This study aimed to examine placental changes in severely anemic women and compare them with the placentae of nonanemic women. The differences thus detected were then correlated with poor neonatal outcomes such as low birth weight and prematurity.

Anemia during pregnancy induces chronic hypoxia, resulting in significant structural and functional changes in the placenta. Placentas from anemic mothers often exhibit hypertrophy, with increased weight relative to the fetus, along with infarcts and calcifications caused by inadequate blood flow and areas of necrosis [1,2]. Fibrinoid necrosis, characterized by deposition of fibrin-like material, can obstruct intervillous spaces and damage placental villi, further compromising nutrient and gas exchange [3]. In response to reduced oxygen levels, the placenta shows increased capillary proliferation within the villi, although the overall efficiency of transfer remains impaired. Additionally, vascular changes such as endothelial damage and reduced smooth muscle content can impair blood flow regulation. Chronic villitis, an inflammatory condition of the villi, and an increased presence of syncytial knots, indicative of higher trophoblast turnover and apoptosis, are also more common in anemic pregnancies [4]. Together, these alterations highlight how anemia-induced hypoxia can trigger compensatory but often insufficient adaptations, ultimately affecting placental function.

The changes in the placenta in anemic women may contribute to adverse fetal outcomes such as preterm delivery and low birth weight. A detailed placental examination shows that pathophysiological changes in the placenta occur according to the severity and duration of the disease. It can guide management to improve neonatal outcomes.

## Materials and methods

This prospective observational study was conducted in the labor room of the Department of Obstetrics and Gynaecology along with the Department of Pathology at Pandit Bhagwat Dayal Sharma Post Graduate Institute of Medical Sciences, Rohtak, for one year from October 1, 2023, to September 30, 2024. It included pregnant females with a gestational age of 32 weeks onward who presented to our center in labor. Their blood hemoglobin levels were evaluated using Sahli’s hemoglobinometer and/or a complete hemogram. The subjects were distributed into two groups based on their hemoglobin level: Group A: Hb <7 g/dL; Group B: Hb ≥11 g/dL.

The study excluded cases with associated obstetric complications; Rh-negative pregnancies; intrauterine growth restriction; multiple gestations; medical disorders of pregnancy, including hypertensive disorders, diabetes mellitus, and chronic renal disease; and cases of mild-to-moderate anemia.

After delivery, the placentae were collected and immediately examined grossly. The following parameters were recorded: (1) weight of the placentae using an electronic weighing machine; (2) maximum diameter of the placenta using a rigid measuring tape; (3) umbilical cord length using a rigid measuring tape, presence of any knot in the umbilical cord, and insertion of the umbilical cord, whether central or marginal; and (4) any gross findings, including calcification, fibrosis, and the presence of a retroplacental hematoma, which were marked and tagged. The placentae were then sent for pathological examination in 10% formalin solution. Two sections were taken, i.e., one close to the umbilical cord (A) and one from the periphery (B). Additional sections were taken from pathological areas identified on gross examination. All stained slides were examined under both low-power and high-power objectives of a light microscope, and any microscopic abnormality was recorded.

Fetal parameters were also recorded at the time of delivery.

## Results

The mean±SD value of serum hemoglobin in Group A was 5.80±0.82 g/dL, while in Group B, it was 11.84±0.52 g/dL. This substantial difference highlights the distinct hematological status between the two groups, making the implications of anemia on placental morphology markedly clear.

The mean±SD birth weight (in kilograms) of newborns in Group A and Group B was 2.655±0.525 and 3.077±0.482, respectively. There was a statistically significant difference between the two groups in terms of mean birth weight (p=0.0001). Fifty-five percent of women in Group A and 65% of women in Group B delivered term babies, showcasing a statistical difference (p=0.361). Both groups had similar APGAR scores at one minute (6.70±0.723) and five minutes (8.70±0.723).

Analysis of placental characteristics revealed that placental weight demonstrated a significant difference, with Group A showing a mean of 442.05±85.23 g compared to 492.40±109.47 g in Group B (p=0.024). The mean maximum placental diameter among participants was 13.75±1.64 cm, with no significant difference between Group A (14.12±1.90 cm) and Group B (13.57±1.33 cm; p=0.139) (Table 1).

**Table 1:** Comparing placental measurements in both groups (significant p-value: p<0.05) The statistical test used was the unpaired t-test.

Gross examination of the placenta	Group A (mean±SD)	Group B (mean±SD)	P-value	Total (mean±SD)
Maximum placental diameter (cm)	14.12±1.90	13.57±1.33	0.139	13.75±1.64
Placental weight (in grams)	442.05±85.227	492.40±109.47	0.024	456.56±100.08
Umbilical cord length (cm)	32.85±4.912	42.35±3.54	0.001	37.03±6.35

Umbilical cord length was also significantly greater in Group B (42.35±3.54 cm) than in Group A (32.85±4.91 cm; p=0.001). Gross calcification was observed in five placentae from Group A but was absent in Group B, a statistically significant finding (p=0.021). Similarly, gross fibrosis was more frequent in Group A (20%) compared to Group B (5%), with statistical significance (p=0.043) (Table 2).

**Table 2:** Comparison of placentae from both groups on gross examination (significant p-value: p<0.05) The statistical test employed is the chi-square test.

	No. of placentae in Group A	No. of placentae in Group B	χ² value	P-value	Total no. of placentae
Central umbilical cord	37 (92.5%)	40 (100%)	3.14	0.077	77
Marginal umbilical cord	3 (7.5%)	0 (0%)	3.14	-	3
Umbilical cord knots	2 (5%)	0 (0%)	2.07	0.152	2
Gross calcification	5 (12.5%)	0 (0%)	5.33	0.021	5
Gross fibrosis	8 (20%)	2 (5%)	4.09	0.043	10
Retroplacental hematoma	9 (22.5%)	4 (10%)	2.29	0.13	13

Histopathological examination of the placentae revealed significant differences between the two groups. Fibrinoid deposition was observed in 47.5% of Group A participants compared to 12.5% in Group B, a statistically significant finding (p=0.001). Syncytial knots were more frequent in Group A (45%) than in Group B (30.76%), with statistical significance (p=0.016). Microscopic calcification was present in 25% of Group A placentae versus 7.5% in Group B, also reaching statistical significance (p=0.034). Increased vascularity was noted in 40% of Group A compared to 22.5% of Group B. Villitis was observed in 20% of Group A and 12.5% of Group B. Microscopic hemorrhage was more common in Group A (32.5%) than in Group B (17.5%), while microscopic infarction was identified in 15% of Group A and 10% of Group B. Although differences in vascularity, villitis, hemorrhage, and infarction did not reach statistical significance, the overall pattern indicates a higher prevalence of pathological changes in Group A compared to Group B (Table 3).

**Table 3:** Comparison of microscopic findings in placentae from both groups (significant p-value: p<0.05) The statistical test employed was the chi-square test.

Parameters	No. of placentae in Group A	No. of placentae in Group B	χ² value	P-value	Total no. of placentae
Fibrinoid deposition	19 (47.5%)	5 (12.50%)	10.45	0.001	24
Syncytial knots	18 (45%)	8 (30.76%)	5.8	0.016	26
Microscopic calcification	10 (25%)	3 (7.5%)	4.47	0.034	13
Increased vascularity	16 (40%)	9 (22.50%)	2.85	0.091	25
Villitis	8 (20%)	5 (12.50%)	0.83	0.363	13
Microscopic hemorrhage	13 (32.50%)	7 (17.50%)	2.38	0.121	20
Microscopic infarction	6 (15%)	4 (10%)	0.46	0.499	10

The microscopic findings indicate that villitis is significantly associated with lower birth weight (<2.5 kg) (p=0.025). Syncytial knots showed a borderline association with low birth weight (p=0.053). Other findings, including fibrinoid deposition, increased vascularity, microscopic calcification, microscopic hemorrhage, and microscopic infarction, did not show statistically significant differences between the two birth weight groups (Table 4).

**Table 4:** Association of microscopic placental findings with the birth weight of the babies (significant p-value: p<0.05) The statistical test employed was the chi-square test.

	No. of deliveries with ≥2.5 kg of birth weight	No. of deliveries with <2.5 kg of birth weight	χ² value	P-value
Fibrinoid deposition	17 (27.41%)	7 (38.88%)	0.88	0.349
Syncytial knots	18 (29.03%)	8 (44.44%)	3.73	0.053
Increased vascularity	17 (27.41%)	8 (44.44%)	1.88	0.17
Villitis	7 (11.29%)	6 (33.33%)	5	0.025
Microscopic calcification	12 (19.35%)	1 (5.55%)	1.96	0.162
Microscopic hemorrhage	15 (24.19%)	5 (27.77%)	0.09	0.757
Microscopic infarction	8 (12.90%)	2 (11.11%)	0.04	0.839

The microscopic findings reveal that villitis is significantly more common in preterm births (53.12%) compared to term births (16.66%) (p=0.001). Other findings, such as fibrinoid deposition, syncytial knots, increased vascularity, microscopic calcification, microscopic hemorrhage, and microscopic infarction, showed no statistically significant differences between term and preterm births (Table 5).

**Table 5:** Association of microscopic placental changes with neonatal prematurity (significant p-value: p<0.05) The statistical test employed was the chi-square test.

	No. of term deliveries	No. of term deliveries	χ² value	P-value
Fibrinoid deposition	14 (29.16%)	10 (31.25%)	1.78	0.182
Syncytial knots	14 (29.16%)	12 (37.5%)	0.61	0.435
Increased vascularity	13 (27.08%)	12 (37.5%)	0.98	0.324
Villitis	8 (16.66%)	17 (53.12%)	10.45	0.001
Microscopic calcification	9 (18.75%)	4 (12.5%)	0.55	0.457
Microscopic hemorrhage	11 (22.91%)	9 (28.12%)	0.28	0.598
Microscopic infarction	6 (12.5%)	4 (12.5%)	0.04	1

## Discussion

The integrity and functionality of the placenta are pivotal in determining fetal growth and developmental outcomes. Anemia reduces maternal hemoglobin levels, thereby impairing oxygen-carrying capacity and diminishing placental perfusion. This hypoxic environment disrupts the placenta’s ability to efficiently transfer oxygen and nutrients while also compromising its endocrine and immunological roles. It directly translates into adverse fetal outcomes in terms of prematurity and low birth weight.

In our study, a slightly higher percentage of preterm deliveries was observed in Group A (45%) compared to Group B (35%), and was similar to the findings of Parks S. et al., who found that 27.1% of severely anemic women delivered prematurely as opposed to 11.6% of non-anemic women (p-value <0.0001) [5].

Our findings demonstrate that neonates born to anemic mothers had significantly lower birth weights compared to those born to non-anemic mothers, with a higher proportion of low-birth-weight infants observed in the anemic group. This pattern is consistent with earlier studies: Biswas et al. (2014) reported mean birth weights of 2.376 kg in the anemic group versus 2.595 kg in the non-anemic group (p=0.01), while Ramteke et al. (2018) found similar differences (2.514±0.179 kg vs. 2.818±0.198 kg, p=0.0001) [6,7]. More recently, Liu et al. (2022) also documented lower mean birth weights among neonates of anemic mothers (2.988±0.549 kg vs. 3.220±0.409 kg) [8]. Our present study aligns closely with these observations, reinforcing the evidence that maternal anemia adversely affects fetal growth. Furthermore, Rahman et al.’s meta-analysis concluded that maternal anemia significantly increases the risk of low birth weight and preterm birth, underscoring its importance as a public health concern [9].

In our study, villitis was significantly associated with low birth weight (p=0.025) and preterm deliveries (p=0.001). These results suggest that inflammation, particularly villitis, may play a role in low birth weight and prematurity. This finding is supported by a study conducted by Lee et al. in 2013, which found that chorioamnionitis is the most common placental pathology associated with preterm birth [10].

Syncytial knots and increased vascularity were also more frequent in both lower birth weight cases and preterm cases, but these differences were not statistically significant. Other factors, such as fibrinoid deposition, microscopic calcification, microscopic hemorrhage, and microscopic infarction, also showed no significant differences between the two groups (p>0.05).

Nigam et al. noted that placental calcification and fibrin deposition were observed significantly more frequently in the placentae of low-birth-weight babies (p<0.01) [11]. Dash S. et al. also observed that the incidence of fetal growth restriction (FGR) was notably higher with placental calcification [12].

Our study identified several significant findings but was limited by its single-center design, small sample size, absence of long-term follow-up of neonates, and exclusion of immunohistochemistry from the histopathological examination.

## Conclusions

Our study found significant changes in the placentae of severely anemic women in terms of an increased incidence of syncytial knots, placental fibrosis, placental calcifications, and villitis, as well as a reduction in placental weight and cord length. Placental fibrosis, calcification, and villitis were associated with adverse fetal outcomes such as preterm delivery and low birth weight. Therefore, a thorough placental examination is essential for all patients with severe anemia.

Patients with severe anemia should deliver at a tertiary care center, as there is an increased risk of both maternal and neonatal complications. Close follow-up of infants born to anemic mothers is recommended to further understand the implications of chronic hypoxia during the antenatal period due to severe anemia.
